# Single-Cell and Bulk Transcriptomics Uncover the Cellular Ecosystem of Vascular Invasion in Intrahepatic Cholangiocarcinoma

**DOI:** 10.3390/cells15111016

**Published:** 2026-05-31

**Authors:** Jianing Fan, Meng Tong, Yunkun Lu, Qianqian Wang, Yangyang Xie, Kainan Lin, Junjie Xu, Xiujun Cai, Xiao Liang

**Affiliations:** 1 Zhejiang Key Laboratory of Multi-Omics Precision Diagnosis and Treatment of Liver Diseases, Department of General Surgery, Sir Run-Run Shaw Hospital, Zhejiang University School of Medicine, Hangzhou 310016, China; 12518629@zju.edu.cn (J.F.); tongmeng9639@163.com (M.T.); kevenloo1@zju.edu.cn (Y.L.); wangqian_cc@zju.edu.cn (Q.W.); 12318549@zju.edu.cn (Y.X.); lkn645916473@163.com (K.L.); walter235@zju.edu.cn (J.X.); 2 Zhejiang Minimal Invasive Diagnosis and Treatment Technology Research Center of Severe Hepatobiliary Disease, Zhejiang Research and Development Engineering Laboratory of Minimally Invasive Technology and Equipment, Hangzhou 310016, China; 3 Zhejiang University Cancer Center, Hangzhou 310058, China; 4 Liangzhu Laboratory, Zhejiang University Medical Center, Hangzhou 311121, China; 5 School of Medicine, Shaoxing University, Shaoxing 312000, China; 6 School of Basic Medical Sciences and Forensic Medicine, Hangzhou Medical College, Hangzhou 310000, China

**Keywords:** intrahepatic cholangiocarcinoma, vascular invasion, hypoxia, tCAFs, *SLC2A1*

## Abstract

Intrahepatic cholangiocarcinoma (ICC) is an aggressive liver malignancy with a rising global incidence and limited therapeutic options. Vascular invasion (VI) is a hallmark of advanced disease, correlating with early recurrence and dismal prognosis, yet its tumor microenvironment (TME) drivers remain elusive. We analyzed single-cell RNA sequencing (scRNA-seq) data from 25 ICC samples to systematically characterize the cellular composition and molecular features related to VI. By integrating bulk RNA-seq data, spatial transcriptomics, and multiplex immunofluorescence, we identified a distinct subset of tumor-like cancer-associated fibroblasts (CAFs), termed tCAFs, enriched in VI-positive tumors. Functional enrichment analyses revealed that tCAFs were prominently associated with hypoxia and angiogenesis pathways, findings corroborated by the significant upregulation of tCAF markers (*MME* and *NT5E*) in ICC-derived CAFs under hypoxic conditions in vitro. Cell–cell communication analysis and spatial mapping uncovered that tCAFs might promote VI primarily through VEGF signaling interactions with endothelial cells. Integrative bioinformatics and RT-qPCR validation identified three key functional genes in tCAFs: *SLC2A1*, *PTGS2*, and *PLOD2*. In endothelial sprouting assays, pharmacological inhibition of *SLC2A1* exerted a pronounced suppressive effect. Consistently, sprouting assays using ICC-derived CAFs with *SLC2A1* knockdown confirmed that its downregulation significantly reduced endothelial sprouting capacity. Importantly, administration of the *SLC2A1* inhibitor BAY-876 effectively suppressed tumor progression and intrahepatic metastasis in the orthotopic ICC mouse model. Our findings define a VI-associated cellular ecosystem and molecular landscape in ICC, unveiling a novel hypoxia–tCAFs–endothelial cells axis. Furthermore, we identify *SLC2A1* as a clinically relevant therapeutic target, offering new insights into tumor VI.

## 1. Introduction

Intrahepatic cholangiocarcinoma (ICC) is the second most common primary liver tumor, and its incidence has shown a significant upward trend in recent years. For the majority of patients, it remains a highly lethal malignant tumor which might be attributable to specific etiological factors, including primary sclerosing cholangitis, hepatolithiasis, and choledochal cysts [1,2,3]. Although surgical resection, gemcitabine and cisplatin-based combination chemotherapy, and immunotherapy are considered potentially effective treatment modalities, the survival period remains short for patients presenting with locally unresectable or metastatic disease [2,4,5,6]. ICC is characterized by profound molecular and cellular heterogeneity, which poses major challenges for accurate classification and effective treatment. Increasing evidence confirms that the tumor microenvironment (TME) plays a critical role in ICC progression, invasion, and therapeutic resistance [7,8].

Dysfunction of the hepatic vascular system represents a central pathological feature of multiple liver diseases, and the concept and application of liver vasomics were first proposed internationally by Qi et al. [9]. Angiogenesis and vascular invasion (VI) are widely recognized as playing crucial roles in the processes of tumor growth, invasion, and metastasis [10]. In hepatocellular carcinoma (HCC), microvascular invasion (MVI) has been established as a key indicator for assessing tumor aggressiveness, postoperative recurrence risk, and prognosis [11,12]. Li et al. delineated a comprehensive single-cell atlas of MVI in HCC and identified its key molecular characteristics through utilizing multi-omics approaches [12]. VI in ICC is primarily characterized by the involvement of the portal vein and hepatic venous systems. It is recognized as one of the critical predictive factors for adverse clinical outcomes in ICC, including reduced overall survival (OS), shortened disease-free survival (DFS), and early recurrence [13,14]. Therefore, investigating the specific mechanisms underlying VI in ICC holds significant clinical implications for tumor treatment and management.

Single-cell RNA sequencing (scRNA-seq) has emerged as a pivotal tool for investigating the tumor microenvironment (TME) and dissecting intratumoral heterogeneity [15,16,17]. Cancer-associated fibroblasts (CAFs) represent a central cellular component of the TME in solid tumors, constituting the peritumoral stromal compartment and exerting profound influences on tumor progression and metastasis [18]. Characterization of canonical CAF subsets, together with the identification of novel CAF subsets, has progressively refined the single-cell CAF landscape. Niu et al. identified a lipid-laden CAF subset marked by *ABCA8a* in pancreatic cancer and demonstrated metabolic interactions between CAFs and tumor cells [19]. Furthermore, a multi-omics study in HCC revealed that interactions between *SPP1*^+^ macrophages and CAFs contribute to the formation of a tumor immune barrier, thereby limiting the efficacy of immunotherapy [20]. Collectively, these findings offer important conceptual and methodological guidance for the investigation of CAF biology. Based on the cellular heterogeneity and dense stromal response of ICC, elucidating the specific functions of CAFs is of critical importance [21,22]. Zhang et al. utilized scRNA-seq to characterize the tumor heterogeneity of ICC and highlighted the interactions between tumor cells and vascular CAFs (vCAFs) [23]. Song et al. classified ICC using two markers, *S100P* and *SPP1*, thereby expanding the understanding of the diversity of ICC tumor cells [24]. Silvia Affo et al. focused on the origins and diverse functions of CAFs in ICC through scRNA-seq [25]. In summary, several pioneering scRNA-seq studies have primarily concentrated on exploring the diversity and heterogeneity of the TME in ICC. However, there remains a lack of comprehensive characterization of the differences in the TME between VI+ (VI positive) and VI− (VI negative) ICC patients at single-cell resolution, as well as the potential molecular mechanisms underlying the development of VI in ICC.

This study first explored the heterogeneity of the TME between VI+ and VI− ICC patients and constructed a comprehensive single-cell atlas. The results revealed that CAFs were enriched in the TME of VI+ ICC, among which the key subset tumor-like CAFs (tCAFs) were identified. The real existence of tCAFs was verified through spatial transcriptomics and multiplex immunofluorescence analysis. Furthermore, potential therapeutic targets were determined through the interactions between tCAFs and endothelial cells, and the molecular mechanism of VI was analyzed and validated through both in vitro endothelial sprouting assays and an in vivo orthotopic ICC mouse model.

## 2. Method

### 2.1. Preprocessing and Analysis of scRNA-Seq Data

Raw scRNA-seq reads from a previous study (Xue et al.) [17] were aligned and quantified using the Cell Ranger (v.3.1) pipeline, with the reference genome being GRCh38. The Seurat workflow (v5.1.0) with default parameters was utilized for downstream analyses. Quality control measures were taken to exclude cells. The specific assessment included three metrics: (1) The number of total UMI count per cell was below 60,000; (2) The number of detected genes was above 500 and below 10,000; (3) The percentage of mitochondrial genes was below 25, and that of hemoglobin genes was below 1. After quality control, the data were normalized. Then, principal component analysis (PCA) was performed using the RunPCA function for dimensionality reduction, and the RunUMAP function was used to visualize the dimensionality reduction results. For cell clustering, we utilized the FindClusters function with a resolution parameter of 0.4.

### 2.2. Cell Clustering and Annotation

We first annotated the 6 major cell types on the basis of well-known marker genes (*CD3E* for T cells, *KLRD1* for NK cells, *EPCAM* for epithelial cells, *LYZ* for myeloid cells, *ACTA2* for fibroblasts, *CD79A* for B cells, and *VWF* for endothelial cells) [17,26]. Among these epithelial cells, malignant epithelial cells were further distinguished from non-malignant cells by inferring large-scale copy-number variations (CNVs) per cell using the inferCNV (v1.21.0) R package.

Next, we conducted the second round of clustering to further identify the subsets of major cell types. Owing to the variable amount and properties of cells in each major cell type, different clustering parameters were adopted. For the clustering of NK/T cells, the resolution was selected as 0.3, and 10 T cell and 2 NK cell clusters were identified. For myeloid cells, the resolution was selected as 0.5, and 1 neutrophil, 8 macrophage, 1 monocyte, 2 dendritic cell, and 1 mast cell clusters were identified. For fibroblasts, the resolution was selected as 0.3, and 9 CAF and 1 pericyte clusters were identified. For endothelial cells, the resolution was selected as 0.3, and 7 subsets were identified.

### 2.3. Tissue Type Enrichment of Cell Subsets

To quantify the enrichment of cell clusters in VI+ and VI− tissues, the index was calculated using the STARTRAC (v0.1.0) R package according to the following formula [27]:

$$R_{o/e}=\frac{Observed}{Expected}$$

in which $R_{o/e}$ is the ratio of observed cell numbers over the expected cell numbers. The expected cell numbers for each combination of cell clusters and tissues were obtained from the chi-squared test. If $R_{o/e}$ > 1, it suggested that one cell cluster was enriched in the specific tissue. If $R_{o/e}$ < 1, it suggested that one cell cluster was depleted in the specific tissue.

### 2.4. Cell–Cell Interactions

To investigate the cell–cell interactions among various cell clusters, we used the Cellchat (v1.6.1) R package for cell communication analysis [28]. We extracted cell clusters of concern and created objects on the basis of the VI+ group and VI− group. Then, the intensity of cellular interactions, the determination of signal sending and receiving cell clusters, and the specific signal pathways were analyzed. Furthermore, based on a pre-existing ligand–receptor interaction database, ligands and receptors significantly overexpressed in specific cell subpopulations were identified. The netVisual_bubble function was utilized to display detailed interaction pairs and probabilities between cell clusters, with a focus on those ligand–receptor pairs that were significantly upregulated in the VI+ group compared with the VI− group.

### 2.5. Gene Set Variation Analysis (GSVA)

To verify the accuracy of the cell clustering result and conduct subsequent survival analysis, GSVA analysis was performed utilizing bulk transcriptome data and the significantly upregulated gene sets of specific cell clusters. For CAF and endothelial cell subsets, we selected the top 20 significantly upregulated genes as representative gene sets; For NK/T and myeloid cell subsets, we selected the top 10 significantly upregulated genes as representative gene sets. Subsequently, the gsva function was utilized for scoring, and the grouping heat map was drawn.

### 2.6. Spatial Transcriptomic Sequencing and Library Construction

The freshly collected ICC tissues were dissected into appropriately sized blocks, and surface residual liquid was absorbed using cleanroom wipers. The tissues were then embedded in OCT Medium (SAKURA, cat.no. 4583), snap-frozen on dry ice, and stored at −80 °C. OCT-embedded samples were sent to Shanghai OE Biotech Co., Ltd. (Shanghai, China) for spatial transcriptomic library preparation, sequencing, and data analysis. Briefly, frozen sections of 10 μm thickness were prepared using a Leica CM1950 Microtome Cryostat (Leica Microsystems, Wetzlar, Germany) at −20 °C. The sections containing target regions were attached to slides (Sigma-Aldrich, Darmstadt, Germany, cat.no. P0425) and subjected to methanol fixation, H&E staining, imaging, and destaining following the 10× Genomics recommended experimental procedure (CG000763). Probe hybridization, CytAssist-mediated transfer, and library construction were performed using 10× Genomics Visium HD Slides (10× Genomics, Pleasanton, CA, USA, cat.no. PN-2000970) and Visium HD Spatial Gene Expression Reagent kit (10× Genomics, Pleasanton, CA, USA, cat.no. PN-1000675). Finally, the libraries were sequenced on the BGI DNBSEQ-T7 sequencing platform in paired-end mode.

### 2.7. Robust Cell Type Decomposition (RCTD) Analysis

Spatial cell-type deconvolution was performed using the spacexr (v2.2.1) R package to infer the cellular composition within each bin. Reference cell-type profiles were derived based on our analysis of the scRNA-seq dataset from the previous study (Xue et al.) [17]. Specifically, the default parameters were used in the create.RCTD function, except for a minimum number of cells >1 for each cell type and a minimum of unique molecular identifier (UMI) counts >1 per pixel, and the mode parameter was set to “doublet” in the run.RCTD function.

### 2.8. Survival Analysis

Grouping was conducted based on the score of GSVA. According to the optimal cut-off value, patients were divided into high and low-expression groups to evaluate the prognostic value of specific cell clusters. Kaplan–Meier survival curves for overall survival (OS) were plotted using the ggsurvplot function in the R package Survminer (v0.5.1).

### 2.9. Multiplex Immunofluorescence (mIF) Staining

Tissue samples were obtained from Sir Run Run Shaw Hospital and used in accordance with the ethical standards of the institutional ethics committee. Formalin-fixed and paraffin-embedded (FFPE) tissues sectioned to 4 μm were used for mIF. Tissue slides were deparaffinized with xylene and rehydrated through a series of staged ethanol solutions (100%, 95%, and 70%). Then, the slides were treated by microwave to induce antigen retrieval for 15 min. Multiplex immunofluorescence staining was conducted using the TG TSA Multiplex IHC Assay Kit (TissueGnostics Asia-Pacific Ltd., Beijing, China). Primary antibodies used included CD10(1:500, ab256494, Abcam) and α-SMA (1:1000, ab7817, Abcam). We performed co-staining of CD10 and α-SMA, following the markers previously established by Lena Cords et al. to identify tumor-like CAFs (tCAFs) [29]. Horseradish peroxidase (HRP)-conjugated secondary antibody was added dropwise according to the species of the primary antibody. Nuclei were stained with DAPI after all the antigens were labeled. Multispectral images were scanned using the EVOS M7000 Imaging System (Thermo Fisher Scientific, Waltham, MA, USA). Cells of interest were quantified using ImageJ (v.1.54p).

### 2.10. RNA Extraction and RT-qPCR

Total RNAs from ICC cells were extracted using Fast Pure Cell/Tissue Total RNA Isolation Kit (Vazyme, Nanjing, China). The Reverse Transcriptase Kit (Takara, Tokyo, Japan) was used for cDNA synthesis from total RNA. qPCR was performed in triplicate on a LightCycler 480 II Real-Time PCR System (Roche Diagnostics GmbH, Mannheim, Germany) using the SYBR RT-PCR Kit (Takara, Tokyo, Japan). Afterward, the expression levels of 5 gene candidates (*SLC2A1*, *PTGS2*, *EGLN3*, *PLOD2*, and *HK2*) were quantified. Additionally, to investigate the relationship between hypoxia and tCAFs, the expression of tCAFs markers (*MME* and *NT5E*) in ICC-derived CAFs was measured under hypoxic conditions. All gene expression levels were determined by the comparative C_t_ method (2−ΔΔCt) with *GAPDH* as an internal control. Primer sequences used in this study are as depicted in Table S8.

### 2.11. Isolation and Culture of Primary Fibroblasts from Human Cholangiocarcinoma Tissue

The human cholangiocarcinoma tissue was minced into small pieces under sterile conditions using ophthalmic scissors. After washing with phosphate-buffered saline (PBS) (Thermo Fisher Scientific, Grand Island, NY, USA), the tissue was evenly distributed in the culture dish and was maintained in Dulbecco’s modified Eagle’s medium (DMEM) (Thermo Fisher Scientific, Grand Island, NY, USA) containing 10% (*v*/*v*) fetal bovine serum (FBS) (Thermo Fisher Scientific, Grand Island, NY, USA) at 37 °C with 5% CO_2_ conditions to allow tissue attachment. After overnight incubation, the culture supernatant and unattached tissue fragments were discarded. The tissue was gently washed with PBS, and fresh DMEM containing 20% FBS was added. The medium was replaced every 2 days. Once fibroblast-like cells, namely ICC-derived CAFs, began to migrate out, the cells were digested with trypsin at an appropriate time, collected by centrifugation, and the cell pellet was reseeded in a new culture dish.

### 2.12. Cell Culture

HEK 293T cell lines (#CM-0005) and Human Umbilical Vein Endothelial Cells (HUVECs) (#CP-H082) were purchased from Procell (Wuhan, China). Primary ICC-derived CAFs were isolated as described above, with ethical approval for tissue collection. All cells were cultured in DMEM supplemented with 10% FBS and maintained in a humidified incubator at 37 °C with 5% CO_2_. For the subsequent sprouting assays, ICC-derived CAFs and HUVECs were cultured under specific hypoxic conditions.

### 2.13. Plasmid Transfection

Employed strictly as a standardized bioengineering tool due to their exceptionally high transfection efficiency for viral production, 293T cells were used for lentivirus packaging [30]. Cells in good condition during the logarithmic growth phase were used for lentivirus packaging. The cells were plated 24 h before transfection, with the cell density maintained at approximately 80%. A mixture of plasmid loaded with lentivirus short hairpin RNA (shRNA) targeting specific genes (sequence information in Table S10) and packaging plasmids psPAX2 and pMD2G was co-transfected into the cells. After 12 h of incubation in the culture medium, the cells were changed to DMEM containing 20% FBS and cultured for an additional 48 h. The lentiviral particles were harvested and filtered through a 0.45 μm membrane to obtain a viral suspension suitable for cell infection. During infection, the ICC-derived CAF density was maintained at approximately 80%. An appropriate amount of viral suspension was added, and the cells were cultured for 24 h. After 24 h, the medium containing the virus was replaced with fresh culture medium, and the cells were cultured for an additional 48 h. After 48 h of infection, clear fluorescence expression was observed. Three days post-infection, drug selection was performed, and stable cell lines expressing the shRNA were obtained.

### 2.14. Sprouting Assay

HUVECs were employed as a well-established and standardized biological indicator to systematically evaluate the pro-angiogenic capacity of the tumor microenvironment [31,32]. Briefly, HUVECs were resuspended after digestion and centrifugation. Here, 80,000 cells were added to 4 mL of fresh complete medium, and 1 mL of methylcellulose (Sigma-M0512) was subsequently added. Then, 25 µL of the mixed solution was dropped onto a 10 cm^2^ culture dish. The dish was inverted and incubated at 37 °C with 5% CO_2_ conditions for 24 h to allow spheroid formation. Once spheroids were visible under the microscope, they were gently detached and centrifuged at 200× *g* for 5 min to form a pellet. The spheroid pellet was resuspended in 2 mL of methylcellulose. Next, 4 mL of collagen stock solution (CORNING-354236) was mixed with endothelial cell medium (ECM) (ScienCell-34356) on ice. Then, 2 mL of the collagen solution was added to the resuspended spheroids in methylcellulose. The mixture was added to a 24-well plate, with 1 mL per well, and incubated for 30 min at 37 °C with 5% CO_2_ conditions. Afterward, 100 µL of the conditioned medium from ICC-derived CAFs was added to each well, and the plate was placed back into the incubator. To further investigate the involvement of the VEGF signaling pathway, a VEGF antibody (Invitrogen, Carlsbad, CA, USA, cat.no. MA5-13182) was added to the ICC-derived CAF during the hypoxic culture period prior to conditioned medium collection. This specific conditioned medium was then applied to the HUVECs sprouting assay to evaluate the necessity of VEGF in ICC-derived CAFs-induced angiogenesis. After 24 h, the number of sprouts and lengths from each group were observed and quantified.

### 2.15. Animal Model

Plasmids (20 μg PX330-p53, 20 μg pT3-EF1aH KRAS G12D, and 4 μg pCMV-SB-LUC) were delivered to a C57BL/6 mouse (male, 8 weeks old) via hydrodynamic tail vein injection. The mouse was then sacrificed, and the tumor in the liver was collected after 6 weeks. The harvested liver tumors were subsequently cut into fragments of approximately 1 mm^3^ and orthotopically implanted into the livers of recipient C57BL/6 mice. Then, 3 days post-implantation, the mice were randomly assigned to two groups to initiate treatment. The treatment group was administered BAY-876 (MedChemExpress, Monmouth Junction, NJ, USA) via daily oral gavage at a dose of 3 mg/kg for 20 days, whereas the control group received an equivalent volume of normal saline on the same schedule. All animal experiments were approved by the Ethics Committee of Sir Run Run Shaw Hospital. In accordance with institutional ethical guidelines, the maximum permitted tumor diameter was <20 mm, and we confirmed that no tumors exceeded this restriction during the study.

### 2.16. Statistical Analysis

Statistical analyses of experimental data were performed using GraphPad Prism (v.10.0), while sequencing data and matched clinical variables were processed in RStudio (v.4.2.1). For two-group comparisons of continuous variables, such as gene expression levels and pathway scores, the Wilcoxon rank-sum test was applied to provide a robust non-parametric assessment, accounting for the potential non-normal distribution and biological outliers inherent in sequencing and clinical datasets. One-way analysis of variance (ANOVA) was used for comparisons involving multiple groups. Kaplan–Meier survival curves were generated to estimate cumulative survival, and differences between groups were assessed using the log-rank test. All experiments were repeated at least three times with biologically independent samples. Statistical significance was denoted as *, **, ***, ****, and ns, corresponding to *p* < 0.05, *p* < 0.01, *p* < 0.001, *p* < 0.0001, and no significant, respectively.

## 3. Result

### 3.1. Decoding the Cellular Composition Landscape of VI+ ICC Tumors Through scRNA-Seq

**Figure 1:**
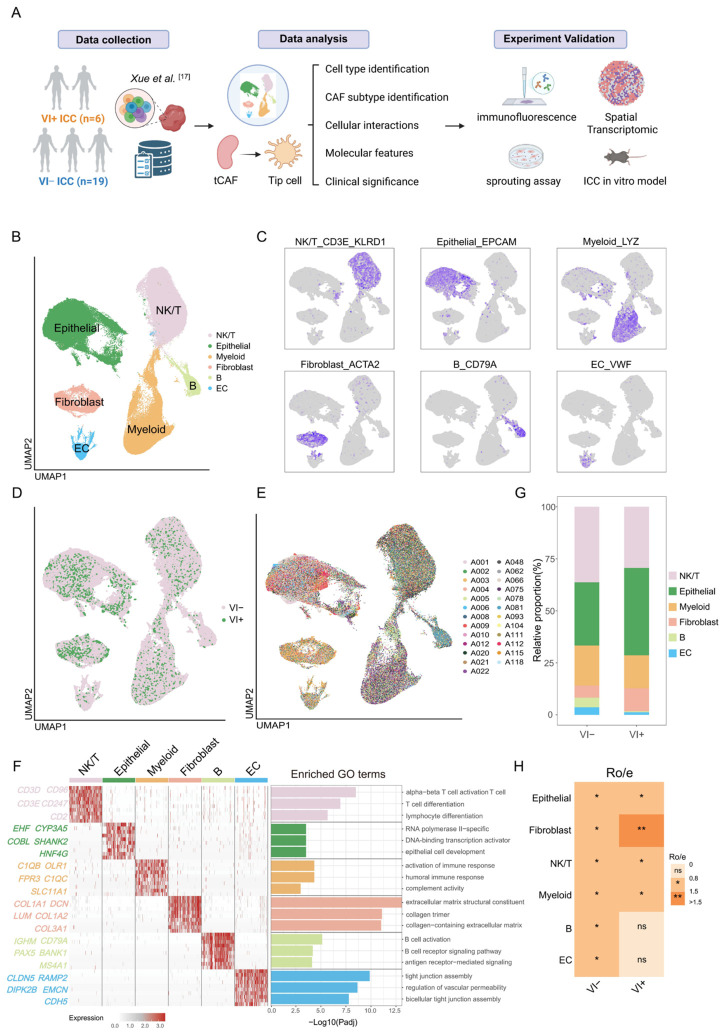
Single-cell transcriptomic analysis of VI+ ICC tumors microenvironment. (**A**) Workflow showing the study design. (**B**) UMAP plot illustrating the 6 major cell types. Colors indicate different cell types. (**C**) UMAP plot illustrating the expression of well-known cell marker genes. (**D**,**E**) UMAP plot illustrating all cells, with colors representing VI+ or VI− tumor tissues and different patients. (**F**) Heatmap showing the top 10 upregulated genes for each cell type, with 5 representative genes marked (left). GO functional enrichment analysis on the basis of the top 10 upregulated genes for each cell type, with 3 representative GO terms (right). (**G**) Cell type proportions in VI+ and VI− ICC tumors. (**H**) Tissue preference of major cell types in VI+ and VI− ICC tumors, revealed by the ratio of observed cell number to expected cell number ($R_{o/e}$) analysis.

VI is considered a critical factor in tumor growth, invasion, and metastasis. However, the TME of ICC exhibiting VI remains poorly understood. To comprehensively dissect the cellular composition and tumor heterogeneity of VI+ ICC tumors, we conducted an in-depth scRNA-seq analysis on 25 ICC specimens (comprising 6 VI+ and 19 VI− samples) utilizing a previously published dataset [17]. The study workflow was illustrated in Figure 1A. Key subsets were first identified through cell clustering and subsequently validated by mIF. Interaction pathways among distinct cellular subsets were then inferred via cell–cell communication analysis, and the functional role of the candidate molecule *SLC2A1* in promoting endothelial sprouting was validated through RT-qPCR and in vitro endothelial sprouting assays. Furthermore, its potential therapeutic efficacy against tumor progression was verified in the orthotopic ICC mouse model using the *SLC2A1* inhibitor BAY-876.

Initially, after data quality control and filtering (Figure S1A), we obtained a total of 169,884 cells. Six major cell types were identified based on canonical marker genes, including 59,545 NK/T cells (*KLRD1* and *CD3E*), 55,159 epithelial cells (*EPCAM*), 31,761 myeloid cells (*LYZ*), 11,394 fibroblasts (*ACTA2*), 6602 B cells (*CD79A*), and 5423 endothelial cells (*VWF*) (Figure 1B,C, Table S2). Subsequently, the distribution of VI+ and VI− characteristics across different cell types was visualized using UMAP plots (Figure 1D). We observed substantial differences in cell types across various tumor samples, reflecting a certain degree of ICC tumor heterogeneity (Figure 1E and Figure S1B). Additionally, the cellular composition landscape was refined based on other clinical features, including relapse state, differentiation, and tumor size (Figure S1C). By identifying the upregulated genes in each cell type and conducting GO functional enrichment analysis, we uncovered corresponding unique biological characteristics, thereby further validating the robustness of the cell-type annotation (Figure 1F, Table S3). The cell proportion and the ratio of observed over expected cell numbers (Ro/e) analyses were performed to discover the distribution preferences of specific cell types in VI+ and VI− ICC tumors. The results indicated that fibroblasts were significantly enriched in VI+ ICC tumors in both analyses (Figure 1G,H, Table S4).

### 3.2. Identification of Major Cell Subsets in VI+ ICC Tumors and the Association of tCAFs with Hypoxic Functions

**Figure 2:**
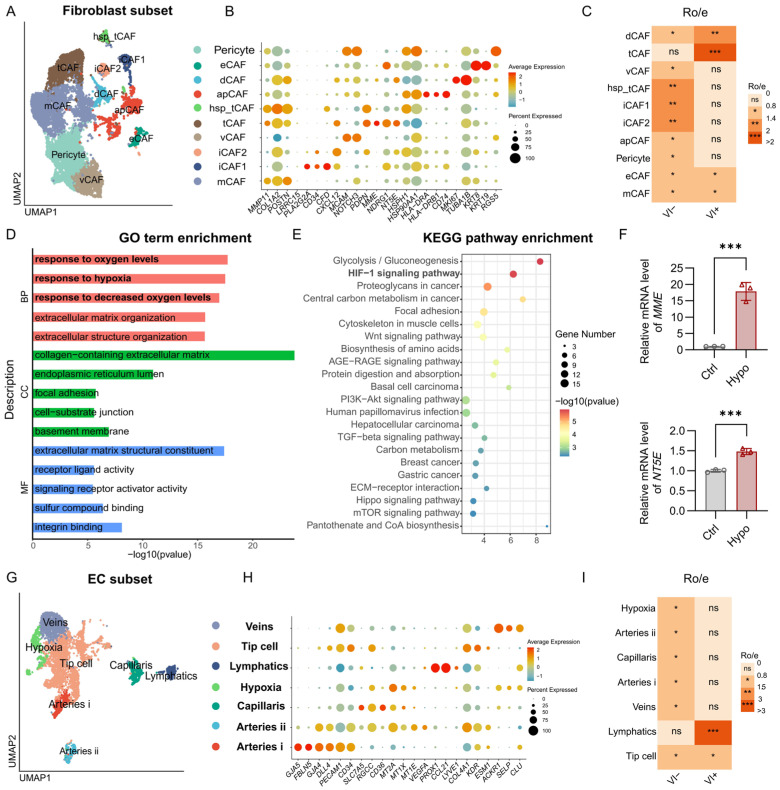
Characterization of fibroblast and endothelial cell subsets in VI+ and VI− ICC. (**A**) UMAP plot illustrating the fibroblast subsets. Colors indicate different cell subsets. (**B**) Dot plot showing the expression of marker genes of each fibroblast subset. (**C**) Tissue preference of fibroblast subsets in VI+ and VI− ICC tumors, revealed by $R_{o/e}$ analysis. (**D**,**E**) GO and KEGG functional enrichment analyses of tCAFs, with bold entries indicating the key biological functions. (**F**) RT-qPCR analysis of *MME* and *NT5E* mRNA levels in ICC-derived CAFs under normoxic (Ctrl) and hypoxic (Hypo) conditions. ***, *p* < 0.001. (**G**) UMAP plot illustrating the endothelial cell subsets. Colors indicate different cell types. (**H**) Dot plot showing the expression of marker genes of each endothelial cell subset. (**I**) Tissue preference of endothelial cell subsets in VI+ and VI− ICC tumors, revealed by $R_{o/e}$ analysis.

Given the notable differences in fibroblast composition between VI+ and VI− tumors, we isolated and subclustered fibroblasts into distinct subsets. A total of 9 distinct subsets of cancer-associated fibroblasts (CAFs) and one pericyte subset were identified (Figure 2A,B). Using the Ro/e analysis to determine the distribution preferences of each CAF subset across different tissues, we found that tumor-like CAFs (tCAFs) and dividing CAFs (dCAFs) were enriched in VI+ ICC tumors, with tCAFs showing the most significant enrichment (Figure 2C). To further investigate the potential functions of tCAFs in ICC, we performed GO and KEGG functional enrichment analyses based on the significantly upregulated genes in tCAFs. The GO analysis revealed that tCAFs were associated with response to oxygen levels, response to hypoxia, and response to decreased oxygen levels (Figure 2D, Table S5). Meanwhile, the KEGG analysis showed significant enrichment of tCAFs in the hypoxia-induced factor-1 (HIF-1) signaling pathway, a classical hypoxia-related pathway (Figure 2E, Table S5). To experimentally corroborate these findings, we cultured ICC-derived CAFs under hypoxic conditions in vitro and examined the expression of tCAFs-specific marker genes. RT-qPCR results revealed that the mRNA levels of *MME* and *NT5E* were significantly elevated after hypoxic exposure (Figure 2F, Table S9). Collectively, these in vitro validations, together with functional enrichment analyses, confirmed a strong correlation between tCAFs and biological characteristics of hypoxia, which might also be a possible way for tCAFs to cause VI of ICC. Building on the established correlation between endothelial cells and vasculature, particularly their roles in angiogenesis, vascular maintenance, and quiescence [33], our study further characterized the subsets of endothelial cells. A total of 7 different subsets of endothelial cells were identified, and we found that lymphatic endothelial cells and tip cells were enriched in VI+ tumors (Figure 2G–I). It has been reported in bladder cancer and breast cancer that the interaction between CAFs and endothelial cells affects the malignant phenotype of tumors [34,35]. Our subsequent study focused on whether there was crosstalk between tCAFs and endothelial cells to promote the VI of ICC.

In addition, to refine the landscape of cell composition in VI+ ICC tumors, we also identified the specific subsets of NK/T and myeloid cells (Figure S2A–D). The Ro/e analysis conducted on the delineated subsets revealed significant enrichment of effector CD8+ T cells, Innate Lymphoid Cells (ILCs), and Liver-resident NK (LrNK) subsets within the NK/T cell cohort in VI+ ICC tumors (Figure S2E). Similarly, the myeloid cell subsets exhibited enrichment of 2 tumor-associated myeloid subsets (TAM_MARCO and TAM_SCL40A1), with the TAM_MARCO subset being notably prominent (Figure S2F). Furthermore, functional enrichment analysis was performed on the TAM_MARCO subset to further elucidate its biological roles, revealing that this subset predominantly regulates the PPAR, IL-17, and HIF-1 signaling pathways (Figure S2G).

Lastly, a chromosomal copy number variation (CNV) analysis was conducted to deduce chromosomal CNV across all epithelial cells. A randomized subsample consisting of 500 NK/T cells and 500 endothelial cells served as the reference (Figure S3A). By evaluating the CNV data and CNV score plots, all epithelial cells were categorized into 10 subsets, among which 8 subsets were identified as malignant epithelial cells (Figure S3B–D). Furthermore, malignant epithelial cells derived from VI+ and VI− ICC tumors were distinguished (Figure S3E). The results of GSVA analysis revealed that VI+ malignant epithelial cells were enriched in a broader spectrum of tumor malignancy-related pathways, including p53 and the Wnt/β-catenin signaling pathways. Notably, a significant enrichment was also observed in hypoxia-related signatures Figure S3F).

Collectively, we identified a distinct CAF subset, termed tCAFs, which was predominantly enriched in VI+ ICC tumors and closely linked to hypoxic features. Therefore, we further validated the presence of tCAFs and investigated their specific biological functions in promoting VI under hypoxic conditions.

### 3.3. Validation of Major Cell Subsets in VI+ ICC Through Bulk RNA-Seq, Spatial Transcriptomics Analysis, and mIF

**Figure 3:**
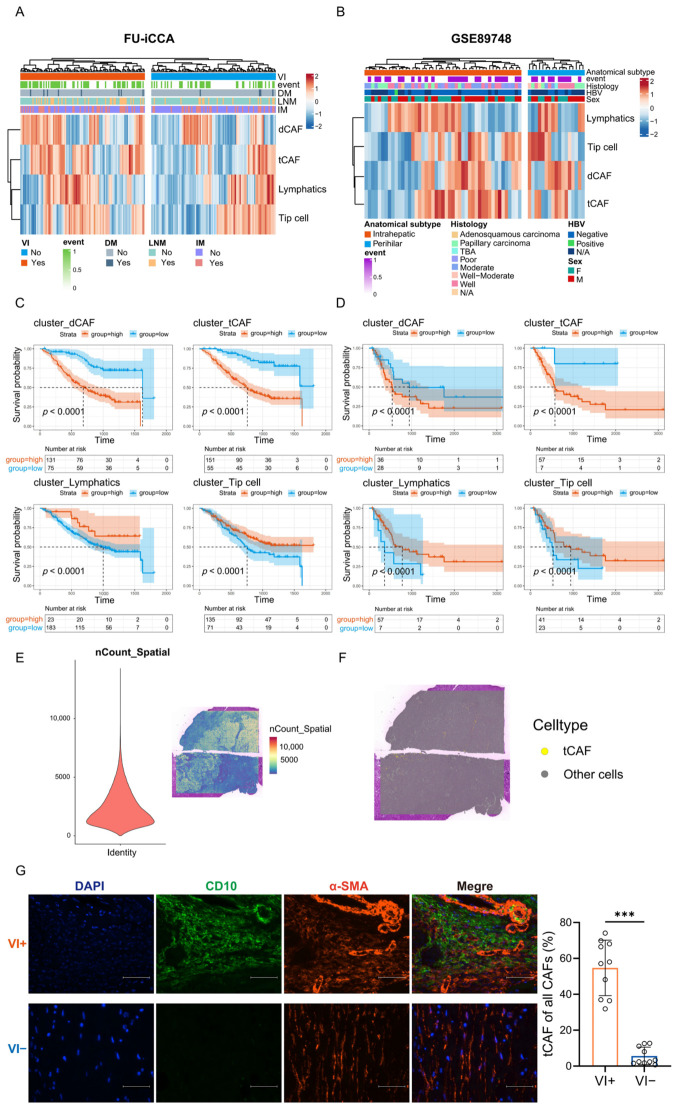
Bulk transcriptomic, spatial transcriptomics analysis, and multiplex immunofluorescence based on major fibroblast and endothelial cell subsets. (**A**,**B**) GSVA heatmap based on bulk RNA-seq data of the FU-iCCA and GSE89748 datasets showing the enrichment scores of the 4 major cell clusters’ signature genes. (**C**,**D**) Kaplan–Meier survival curves based on the FU-iCCA and GSE89748 datasets showing the OS of 4 major cell clusters. (**E**) Visualization of spatial quality control metrics (nCount_Spatial). (**F**) Spatial mapping illustrating the presence and distribution of tCAFs (yellow) within the tumor tissue sections. (**G**) Representative multiplex immunofluorescence staining images indicating tCAFs (CD10+/α-SMA+) in VI+ and VI− ICC samples. Scale bar, 50 μm. The bar plot shows the quantification results. ***, *p* < 0.001.

To validate the reliability of the single-cell clustering results, we verified the key subsets of fibroblasts (tCAFs, dCAFs) and endothelial cells (lymphatic endothelial cells, tip cells) in the FU-iCCA [36] and the GSE89748 [37] datasets. Based on the aforementioned bulk RNA-seq data, GSVA analysis revealed that VI+ ICC patients exhibited higher enrichment scores for the signature genes of the aforementioned cell subsets, defined by the top 20 significantly upregulated genes. Specifically, tCAFs were more enriched in the VI+ group compared to the VI− group, providing additional evidence for the potential relationship between tCAFs and VI (Figure 3A,B). In addition, we conducted prognostic survival analysis on tCAFs, dCAFs, lymphatic endothelial cells, and tip cells based on the above two datasets. The results consistently indicated that patients with high enrichment scores of tCAFs exhibited poorer prognosis, whereas those with high enrichment scores of lymphatic endothelial cells and tip cells demonstrated a favorable prognosis (Figure 3C,D). To further investigate the spatial distribution of tCAFs, we performed spatial transcriptomics analysis on tissue sections from two ICC patients from our center (Sir Run Run Shaw Hospital; clinical characteristics are summarized in Table S1). Spatial mapping clearly demonstrated the presence and distribution of tCAFs within the tumor microenvironment (Figure 3E,F). Multiplex immunofluorescence staining of the tissue samples organized by our center further confirmed the increased abundance of tCAFs (CD10+, SMA-α+) in VI+ ICC samples compared to VI− ICC samples (Figure 3G, Table S6).

Moreover, we validated the single-cell clustering results of NK/T and myeloid cells using bulk RNA-seq data. On the basis of the top 10 significantly upregulated genes in each key subset, GSVA and prognostic survival analyses were performed. As shown in Figure S4, in VI+ ICC patients, the enrichment scores of TAM_MARCO signature genes were higher, and higher scores were significantly associated with worse survival outcomes.

In summary, we validated the reliability of the single-cell results and investigated the clinical prognosis of all key subsets, confirming that tCAFs were associated with worse prognosis of ICC.

### 3.4. Cell–Cell Communication Analysis and Functional Characterization Reveal tCAFs-Tip Cells Crosstalk via VEGF Signaling in VI+ ICC Tumors

**Figure 4:**
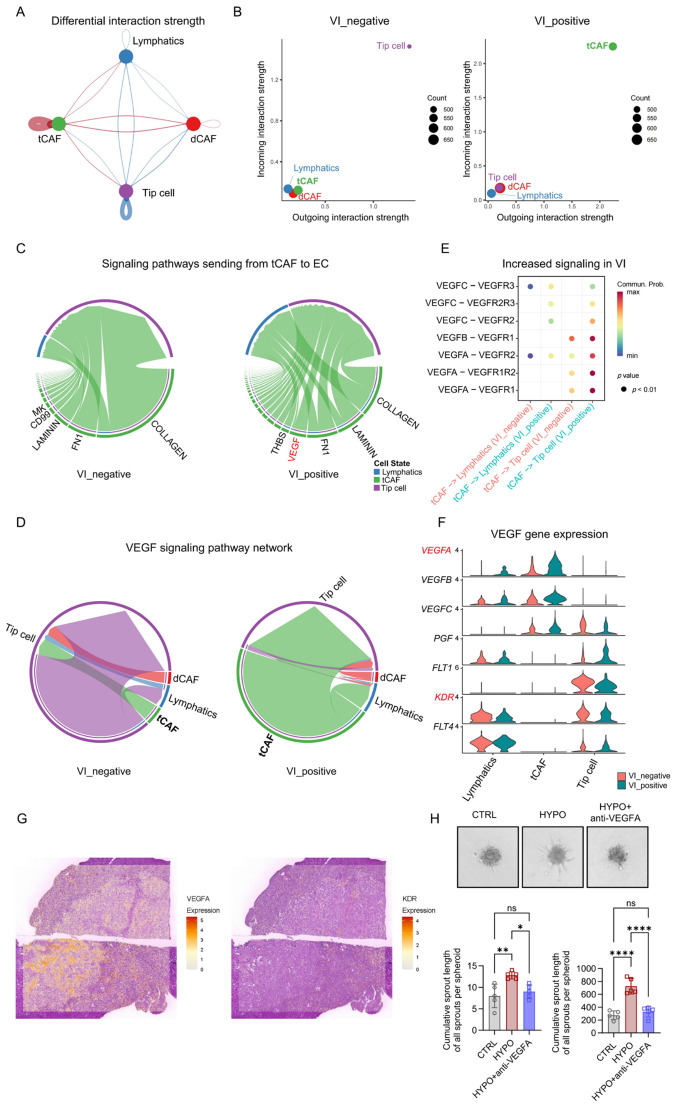
Cell–cell interactions and functional characterization between major fibroblast and endothelial cell subsets. (**A**) Network plot showing the 4 major cell clusters with differential interaction strength. The red line indicates upregulations, with the VI+ group relative to the VI− group, while the blue line is the opposite. The edge width is proportional to the interaction strength. (**B**) Comparing the outgoing and incoming interaction strength of the 4 major cell clusters in 2D space between VI+ and VI− groups. (**C**) Chord plot showing changes in the key signaling pathways from tCAFs to endothelial cell clusters. (**D**) Chord plot showing changes in the interaction relationships of the 4 major cell clusters on the VEGF signaling pathway. (**E**) Bubble plot showing the upregulated ligand–receptor pairs in the VEGF signaling pathway from tCAFs to endothelial cell clusters. (**F**) Expression distribution of the VEGF signaling pathway genes in VI+ and VI− groups. (**G**) Spatial transcriptomic visualization of VEGFA and its receptor KDR expression in ICC tissue sections. (**H**) Representative images and quantification of the HUVECs sprouting assay. Spheroids were treated with conditioned medium from ICC-derived CAFs cultured under normoxia (CTRL), hypoxia (HYPO), or hypoxia supplemented with a VEGFA neutralizing antibody (HYPO + anti-VEGFA). *, *p* < 0.05; **, *p* < 0.01; ****, *p* < 0.0001; ns, not significant.

To investigate how key cell subsets interact to promote VI, we performed comparative analyses of cell–cell communication across VI+ and VI− groups. These analyses elucidated interactions among tCAFs, dCAFs, lymphatic endothelial cells, and tip cells, allowing the identification of critical one-to-one cellular interaction pairs. As shown in Figure 4A, compared to the VI− group, the interaction strengths of tCAFs with dCAFs, lymphatic endothelial cells, and tip cells were upregulated in the VI+ group. To identify the specific cell subsets sending or receiving signals in VI+ and VI− groups, a 2D spatial analysis of outgoing and incoming interaction strengths was performed. The results indicated that in the VI+ group, tCAFs acted as the primary signal-sending cell subset, while the other cell subsets served as signal-receiving cell subsets (Figure 4B). Additionally, we analyzed the signaling pathways through which tCAFs acted on lymphatic endothelial cells and tip cells. The results revealed that the top 3 signaling pathways in both VI+ and VI− groups were COLLAGEN, FN1, and LAMININ. However, the vascular endothelial growth factor (VEGF) signaling pathway was significantly enriched exclusively in the VI+ group (Figure 4C). We further specifically analyzed the VEGF signaling pathway and found that compared to the VI− group, tCAFs predominantly acted on tip cells in the VI+ group. It suggested that tCAFs primarily targeted tip cells within endothelial cell subsets to influence VI of ICC (Figure 4D). The receptor-ligand interactions were identified within the VEGF signaling pathway. In the VI+ group, tCAFs expressed higher levels of *VEGFA* and *VEGFB*, while tip cells expressed higher levels of *FLT4*. Moreover, most VEGF ligand–receptor pairs were significantly upregulated (Figure 4E,F). To validate these findings in a spatial context, we examined the spatial distribution of *VEGFA* and its primary receptor *KDR* using spatial transcriptomics. The result demonstrated that *VEGFA* and its cognate receptor *KDR* exhibited partially consistent spatial distribution patterns (Figure 4G). To further confirm the functional role of tCAFs-derived VEGFA in angiogenesis, we performed an in vitro sprouting assay using conditioned medium from ICC-derived CAFs cultured under hypoxia. The results showed that hypoxia-cultured ICC-derived CAF-conditioned medium significantly promoted HUVECs sprouting, whereas the addition of the VEGFA neutralizing antibody markedly attenuated this pro-angiogenic effect, reducing both sprout number and length (Figure 4H). Collectively, it indicated that tCAFs primarily modulate VI through the VEGF signaling pathway on tip cells.

In addition, we explored interactions between tCAFs and various subsets of NK/T and myeloid cells. In the VI+ group, tCAFs also served as the primary signal-sending cell subset, while TAM_MARCO acted as the main signal-receiving cell subset (Figure S5A,B). We further investigated specific signaling pathways and ligand–receptor pairs, revealing that tCAFs might primarily act on TAM_MARCO through the MIF signaling pathway (Figure S5C–F). MIF has been shown to stimulate tumor cells to overexpress VEGF through activation of the MAPK signaling pathway, thereby increasing tumor microvascular density [38]. Our results provided an alternative potential mechanism underlying VI in ICC.

In conclusion, our study suggested that tCAFs promoted VI by acting on tip cells through the VEGF signaling pathway.

### 3.5. Integrated Bioinformatics and Experimental Validation: Identify Key Functional Gene in tCAFs

**Figure 5:**
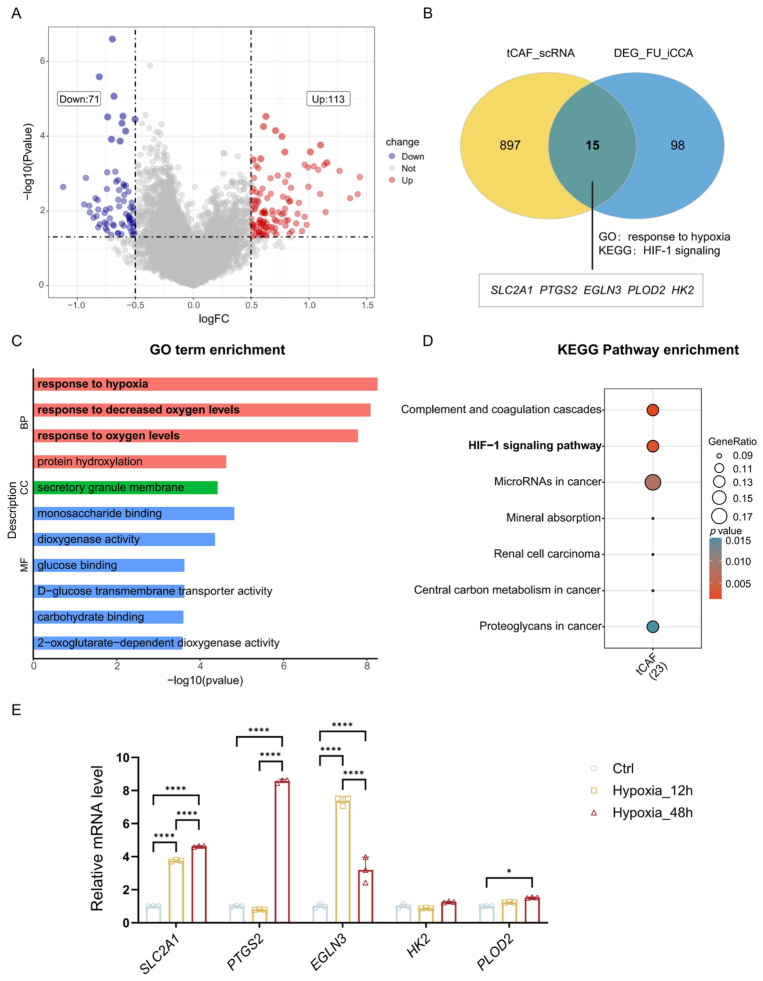
Functional identification and filtering of key genes in tCAFs. (**A**) Volcano plot showing the up- and down-regulated genes after differential analysis of FU-iCCA dataset. (**B**) Venn diagram of upregulated genes in tCAFs and DEGs FU-iCCA dataset. (**C**,**D**) GO and KEGG functional enrichment analyses of key genes, with bold entries indicating the key biological functions. (**E**) qRT-PCR analysis of 5 candidates after control, 12 h of hypoxia, and 48 h of hypoxia. *, *p* < 0.05; ****, *p* < 0.0001.

Firstly, the bulk RNA-seq data of FU-iCCA [36] were stratified into VI-positive and VI-negative groups, and the differential expression gene (DEG) analysis was performed (Figure 5A). Subsequently, we intersected the 113 upregulated DEGs with the 912 significantly highly expressed genes identified in the tCAFs subset from the single-cell data, resulting in a gene list comprising 15 genes (Figure 5B, the gene list: ***SLC2A1***, ***PTGS2***, ***EGLN3***, ***PLOD2***, ***HK2***, *CA12*, *HMGA2*, *STEAP1*, *STEAP2*, *CRABP2*, *IGF2BP2*, *GALNT5*, *CD109*, *TREM1*, *APOL1*). Based on this gene list, GO and KEGG functional enrichment analyses were conducted. The GO analysis revealed significant associations with hypoxia-related functions, including response to hypoxia, response to decreased oxygen levels, response to oxygen levels, and dioxygenase activity (Figure 5C, Table S7). Meanwhile, the KEGG analysis indicated significant enrichment in the HIF-1 signaling pathway (Figure 5D, Table S7). According to the key genes related to hypoxia biological functions and signaling pathways, 5 candidates were identified, including *SLC2A1*, *PTGS2*, *EGLN3*, *PLOD2*, and *HK2* (Figure 5B). To further validate whether these 5 hypoxia-related genes were indeed key functional genes in tCAFs, we performed RT-qPCR to assess their expression levels in ICC-derived CAFs under hypoxic conditions.

Under 12 h and 48 h hypoxic culture conditions of ICC-derived CAFs, compared with the control group, the mRNA expression levels of *SLC2A1*, *PTGS2*, *EGLN3*, and *PLOD2* all increased, while there was no significant difference in *HK2* (Figure 5E, Table S9). EGLN3a is a proline hydroxylase whose primary function is to regulate the HIF factors. It has been shown to be involved in mediating CAF activation and inhibiting HIF-1α [39]. Therefore, subsequent analyses focused on investigating the roles of *PTGS2*, *SLC2A1*, and *PLOD2* in endothelial cell angiogenesis.

Overall, we identified 5 hypoxia-related genes in tCAFs by integrating single-cell and bulk RNA-seq data and confirmed the candidate genes potentially driving VI utilizing RT-qPCR.

### 3.6. SLC2A1 Facilitates Hypoxia-Induced Sprouting of Endothelial Cells

**Figure 6:**
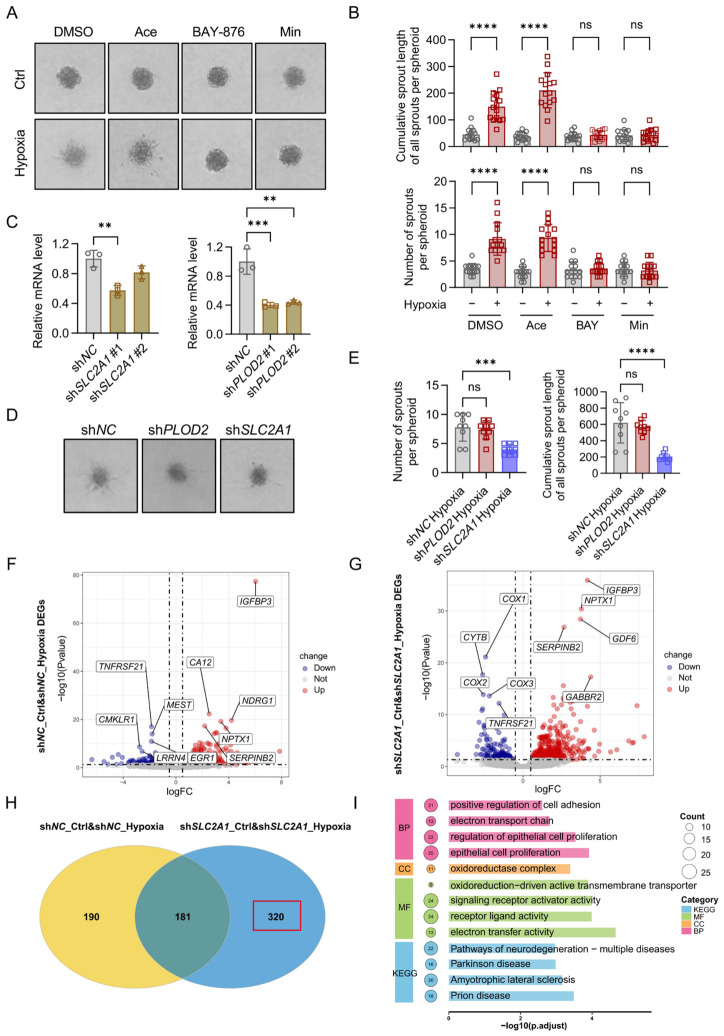
*SLC2A1* promotes the sprouting of endothelial cells under hypoxic conditions. (**A**) Representative plots showing the effects of *PTGS2*, *SLC2A1*, and *PLOD2* inhibitors and DMSO on endothelial cells sprouting (Acetaminophen, inhibitor of *PTGS2*; BAY-876, inhibitor of *SLC2A1*; Minoxidil, inhibitor of *PLOD2*). (**B**) Cumulative sprout length of all sprouts and number of sprouts per spheroid in the inhibitor and control groups. (**C**) mRNA expression levels of sh*PLOD2* and sh*SLC2A1* in ICC-derived CAFs. (**D**) Representative plots showing the effects of conditioned media from sh*PLOD2*, sh*SLC2A1*, and control groups on endothelial cells sprouting. (**E**) Cumulative sprout length of all sprouts and number of sprouts per spheroid in the sh*PLOD2*, sh*SLC2A1*, and control groups. (**F**,**G**) Volcano plots of DEGs in control and sh*SLC2A1* under normal and hypoxic conditions. (**H**) Venn diagram showing the overlap of DEGs between the control and sh*SLC2A1* groups. (**I**) GO and KEGG functional enrichment analyses based on the uniquely expressed genes in the sh*SLC2A1* group. **, *p* < 0.01; ***, *p* < 0.001; ****, *p* < 0.0001; ns, not significant.

Based on the upregulated mRNA expression levels of *PTGS2*, *SLC2A1*, and *PLOD2* under hypoxic conditions, their corresponding inhibitors, Acetaminophen, BAY-876, and Minoxidil, were used to investigate their effects on the sprouting of endothelial cells, as the results shown in Figure 6A. Sprouting of endothelial cells was markedly enhanced under hypoxic conditions, whereas culturing with BAY-876 and Minoxidil significantly suppressed sprouting, suggesting that *SLC2A1* and *PLOD2* might facilitate endothelial cell angiogenesis (Figure 6B). Furthermore, we established stable knockdown ICC-derived CAFs for *SLC2A1* and *PLOD2*, and the corresponding conditioned media were applied to endothelial cells to further evaluate their effects on the sprouting of endothelial cells (Figure 6C,D). The results demonstrated that sh*PLOD2* exerted no significant effect on the sprouting of endothelial cells under hypoxia, while sh*SLC2A1* significantly inhibited sprouting (Figure 6E). This might be due to the fact that minoxidil could simultaneously affect multiple lysyl hydroxylases or indirectly downregulate *PLOD2*, indicating that it was not a specific *PLOD2* inhibitor [40]. In addition, we examined the expression patterns of *PTGS2*, *SLC2A1*, and *PLOD2* across different CAF subtypes. As shown in Figure S6A, *PLOD2* was broadly expressed across multiple CAF subtypes, whereas *PTGS2* showed relatively higher expression mainly in iCAFs. In contrast, *SLC2A1* exhibited a more selective expression pattern and was highly expressed predominantly in tCAFs. Together, these results supported the prioritization of *SLC2A1* as a candidate involved in hypoxia-induced, tCAF-mediated endothelial sprouting.

To further elucidate the potential molecular mechanisms of *SLC2A1* in promoting endothelial sprouting under hypoxic conditions, transcriptome analysis on the control and sh*SLC2A1* CAFs in both normal and hypoxic conditions was performed. We found that the DEGs between control and sh*SLC2A1* groups under normal and hypoxic conditions, respectively, with *IGFBP3* identified as the top commonly upregulated gene in both comparisons (Figure 6F,G). To characterize the unique DEGs specific to the sh*SLC2A1* groups, GO and KEGG functional enrichment analyses were utilized. The results revealed that these 320 DEGs were mainly associated with the electron transport chain, oxidoreductase complex, oxidoreduction-driven active transmembrane transporter, and electron-transfer activity (Figure 6H,I). *SLC2A1*, encoding Glucose Transporter 1 (GLUT1), primarily influences tumor energy metabolism [41], which is consistent with our findings. Meanwhile, we investigated the distinct functions of sh*SLC2A1* under normal and hypoxic conditions, with the DEGs shown in Figure S6B,C. Functional enrichment analysis revealed that the TGF-β signaling pathway was significantly enriched under both normal and hypoxic conditions (Figure S6D,E). Accordingly, the expression levels of genes involved in the TGF-β signaling pathway were analyzed across the different groups. The results demonstrated that sh*SLC2A1* markedly upregulated TGF-β pathway-related genes under hypoxic conditions, which was further supported by GSEA analysis (Figure S6F,G).

### 3.7. SLC2A1 Correlates with Poor Prognosis and Serves as a Promising Therapeutic Target to Suppress ICC Progression and Metastasis In Vivo

**Figure 7:**
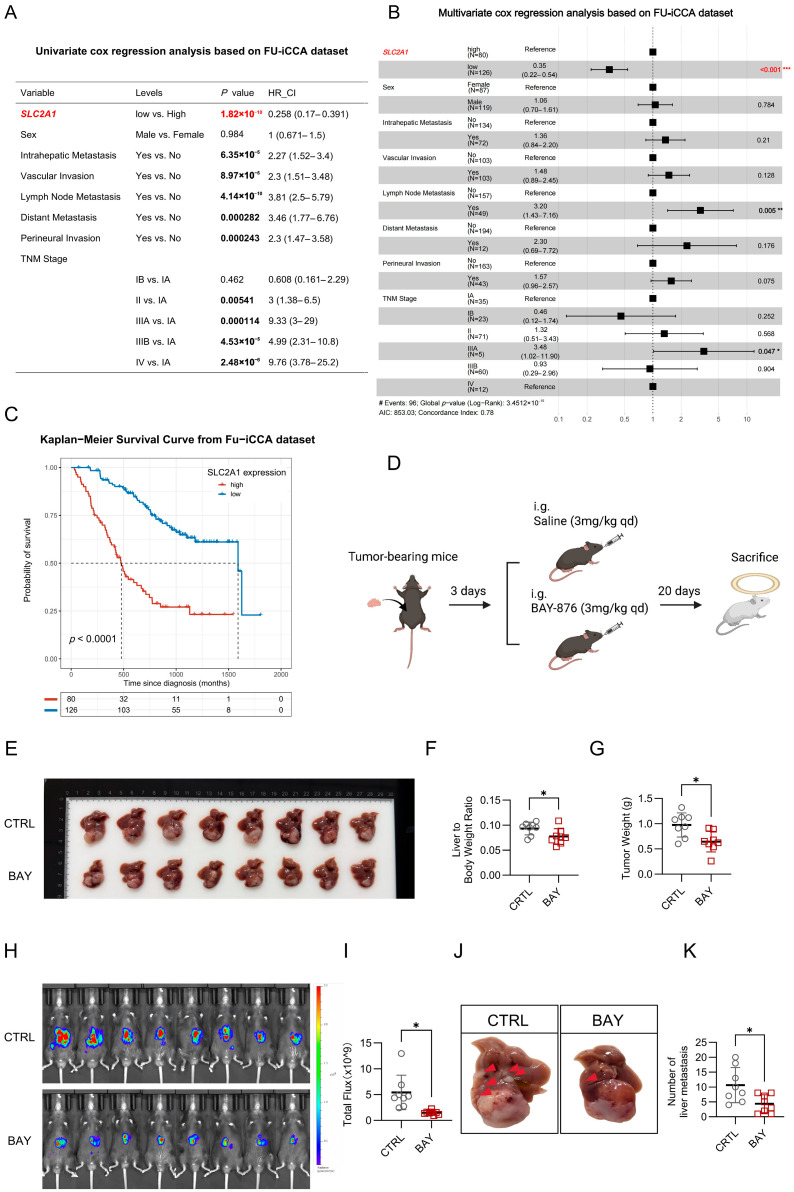
*SLC2A1* correlates with poor prognosis and serves as a potential therapeutic target for ICC. (**A**,**B**) Univariate and multivariate Cox regression analyses of the FU-iCCA cohort identifying *SLC2A1* as an independent prognostic factor for OS. (**C**) Kaplan–Meier survival curves based on the FU-iCCA dataset comparing OS between *SLC2A1*-high and *SLC2A1*-low groups. (**D**) Experimental scheme of the orthotopic ICC mice model and BAY-876 treatment (3 mg/kg, i.g., q.d.). (**E**–**G**) Representative images of harvested livers and tumors (**E**), with statistical quantification of liver-to-body weight ratio (**F**) and absolute tumor weight (**G**). (**H**,**I**) In vivo bioluminescence imaging (**H**) and quantification of total flux (**I**) showing tumor burden. (**J**,**K**) Representative macroscopic images of liver surfaces ((**J**), metastatic nodules indicated by arrowheads) and quantification of intrahepatic metastatic nodules (K). *, *p* < 0.05; **, *p* < 0.01; ***, *p* < 0.001.

To evaluate the clinical significance of *SLC2A1*, we first performed survival analysis using the FU-iCCA cohort. Univariate and multivariate Cox regression analyses identified high *SLC2A1* expression as a robust independent risk factor for poor OS in ICC patients (Figure 7A,B). Kaplan–Meier curves further confirmed that patients with high *SLC2A1* expression had significantly shorter survival times compared to the low-expression group (Figure 7C).

Given its prognostic value, we next investigated the therapeutic potential of targeting *SLC2A1* in vivo using an orthotopic ICC mouse model. Following the implantation of tumor tissues, mice were treated with either saline or the specific *SLC2A1* inhibitor BAY-876 via intragastric administration (Figure 7D). Pharmacological inhibition of *SLC2A1* significantly attenuated the tumor burden, as evidenced by a marked reduction in liver-to-body weight ratio and absolute tumor weight (Figure 7E–G). These findings were consistent with the results of the in vivo bioluminescence imaging, which showed that the total light flux in the BAY-876 treatment group was significantly lower than that in the control group (Figure 7H,I). Crucially, we observed that *SLC2A1* inhibition significantly suppressed intrahepatic metastasis. The number of metastatic nodules in the liver was markedly reduced in the BAY-876 treatment group compared to the control group (Figure 7J,K). Collectively, these results demonstrated that *SLC2A1* was not only a critical prognostic indicator but also a potential viable therapeutic target for inhibiting ICC progression and vascular-mediated metastatic spread.

In conclusion, these findings demonstrate that hypoxia-driven remodeling of the tumor microenvironment in ICC fosters the emergence of a distinct CAF subset–termed tCAFs, which exhibits pro-angiogenic crosstalk with endothelial cells. Mechanistically, we identify *SLC2A1* (GLUT1) as a critical metabolic regulator that potentiates endothelial sprouting and VI, thereby exacerbating ICC progression. Our study unveils a hypoxia–tCAF–endothelial cell signaling axis as a potential therapeutic target for disrupting tumor vascularization in ICC.

## 4. Discussion

Despite significant advances in the treatment of ICC, the OS of advanced patients remains poor. VI, recognized as an important prognostic factor in the latest ICC staging system, is closely associated with patient survival and tumor recurrence [42,43]. In this study, a comprehensive characterization of the TME differences between VI+ and VI− ICC was provided by single-cell analysis. Through integrating bulk transcriptomic, spatial transcriptomics, and mIF staining, we identified distinct cell subsets specifically enriched in VI+ ICC and key molecular features that contribute to VI.

In particular, we identified a specific CAF subset in VI+ ICC, tCAFs, characterized by significantly differential expression of genes associated with proliferation, migration, metastasis, and stress response. Moreover, this cluster specifically expressed high levels of membrane metalloendopeptidase (*MME*), encoding CD10, which is typically expressed in tumor cells but also serves as a characteristic highly expressed gene for this specific CAF subset. This is further supported by the hypoxia marker carbonic anhydrase IX (CAIX), suggesting that these cells might originate from hypoxic regions of the tumor [29]. Previous studies have reported that hypoxic tCAFs are enriched in lung squamous cell carcinoma (LUSC) and are closely related to chemoresistance, high tumor grade, and poor patient prognosis [44,45]. In breast cancer, tCAFs have been found to display a close spatial relationship with tumor cells, implying potential reciprocal interactions. To further validate tCAFs at the protein level in our study, we utilized CD10 and α-SMA as co-staining markers, consistent with the methodology established by Lena Cords et al. in identifying specific fibroblast niches [29]. Notably, our identified tCAFs should be distinguished from the CD10+ GPR77+ CAFs previously reported by Su et al. in breast and lung cancer [46]. While Su et al. demonstrated that their CAF subset provides a niche for cancer stem cells to promote tumor progression and chemoresistance, tCAFs identified in our study were primarily characterized by the high expression of *MME*, *NT5E*, and *NDRG1*, and were intrinsically linked to hypoxia-related functions. Furthermore, it was significant to distinguish tCAFs from the CD146-positive vascular CAFs (vCAFs) identified by Zhang et al. in ICC [23]. While vCAFs promoted ICC progression through secreting high levels of interleukin-6 (IL-6) to induce significant epigenetic alterations in tumor cells, tCAFs in our study exhibited a distinct functional profile by primarily responding to hypoxia signaling and interacting with endothelial cells via VEGF signaling to drive VI. Overall, our study firstly expanded the functional roles of tCAFs in ICC. We found that tCAFs were significantly enriched in VI+ ICC, exhibiting activation of the HIF-1 and VEGF signaling pathways and hypoxia-responsive features, which were consistent with the gene signatures of tCAFs. Additionally, recent neuro-oncology studies have revealed tCAFs as a key player in the perineural invasion of pancreatic cancer. Whether this occurs through attenuation of oxidative stress in peritumoral nerves remains to be elucidated [47].

Angiogenesis, immune regulation, and extracellular matrix remodeling are among the major biological features enriched in tumor endothelial cells (TECs) [48]. Through cell–cell communication analysis, our study demonstrated significant interactions between tCAFs and endothelial cells in ICC. By integrating bulk transcriptomic data, we further identified a gene list strongly associated with hypoxia and VI. Among these, *SLC2A1* emerged as the most critical molecule, directly influencing the sprouting of endothelial cells. *SLC2A1*, which encodes GLUT1, serves as the rate-limiting transporter for glucose uptake and has been shown to modulate metabolic reprogramming in several cancers, including colorectal cancer, lung cancer, and hepatocellular carcinoma, thereby contributing to tumor progression [49,50,51]. Recent studies in ICC have found that epithelial cells with high *SLC2A1* expression and CAF with high *VEGFA* expression were enriched in the CA199-positive group, revealing that hypoxia-induced metabolic reprogramming might promote the formation of these cell subsets. Their cell–cell communications were thought to enhance tumor angiogenesis [52]. In our study, co-culture experiments using *SLC2A1* inhibitors or conditioned media from *SLC2A1*-knockdown CAF demonstrated that sprouting of endothelial cells was significantly suppressed under hypoxic conditions. Functional enrichment analysis further revealed that *SLC2A1* might regulate sprouting through metabolic pathways, particularly those related to the electron transport chain and oxidoreductase complexes, consistent with its metabolic role. It would be of vital interest to explore whether *SLC2A1* plays key roles through the canonical glucose transport function. Additionally, we evaluated the therapeutic potential of targeting *SLC2A1* in vivo. Our results demonstrated that administration of the *SLC2A1*-specific inhibitor BAY-876 significantly suppressed tumor progression in the orthotopic ICC mouse model. Crucially, the inhibition of *SLC2A1* led to a marked reduction in intrahepatic metastasis. Given that intrahepatic metastasis in ICC primarily occurs via hematogenous spread, where VI serves as the indispensable initial step, these findings reinforce the role of the *SLC2A1* in driving the early stages of the metastatic cascade.

Deciphering the malignant phenotypes of ICC using single-cell transcriptomics is of paramount importance. While tumor cells are generally regarded as the primary drivers of malignant progression, other components within the TME also exert specific biological functions. Xun et al. elucidated the spatiotemporal landscape of intrahepatic metastasis in ICC. They identified a population of metastatic COL3A1+ epithelial cells and revealed that these cells promoted ICC invasion by inducing endothelial-to-mesenchymal transition (EndMT) [53]. In contrast, our study highlighted tCAFs as pivotal drivers of VI. We demonstrated that tCAFs interacted with endothelial cells via the VEGF signaling pathway under hypoxic conditions, thereby complementing the existing knowledge regarding the role of CAFs in shaping ICC malignant phenotypes. Similarly, Liao et al. identified an aggressive basal-like tumor cell subset associated with poor prognosis [54]. We indicated that tCAFs were also correlated with adverse survival outcomes in ICC patients. By integrating bulk transcriptomic data, *SLC2A1* was identified as a clinically relevant therapeutic target. Together with our detailed characterization of myeloid cells, NK/T cells, and epithelial cells, our findings refined the comprehensive landscape of the TME specific to VI in ICC. Furthermore, a recent study reported by Fan et al. demonstrated that MARCO+ tumor-associated macrophages (TAM_MARCO) represent the predominant macrophage subset in ICC, characterized by enhanced angiogenesis and hypoxia [55]. Our data found that TAM_MARCO was significantly enriched in VI+ ICC, and functional analyses revealed a strong association with the HIF-1 signaling pathway. Moreover, TAM_MARCO exhibited intensive cellular interactions with tCAFs in VI+ ICC, potentially promoting VI through the MIF-CD74 axis. Consistently, a single-cell study investigating microvascular invasion (MVI) in HCC also identified an upregulated MIF-CD74 axis within stromal-immune interactions in MVI+ HCC [12]. Moreover, MIF has been shown to directly bind the CD74 receptor on endothelial cells to facilitate angiogenesis in breast cancer. These findings provide a novel perspective for understanding the biological mechanisms underlying VI in ICC.

In conclusion, this study provides the first comprehensive delineation of the cellular ecosystem and molecular characteristics associated with VI in ICC. We identified specific cellular subsets that are tightly linked to enhanced VI and poor prognosis. Notably, our analyses revealed intensive intercellular communication networks, revealing the hypoxia–tCAFs–endothelial cells signaling axis and underscoring critical molecules that potentially drive the onset of VI in ICC. Nevertheless, several limitations should be acknowledged. First, the relatively small and imbalanced sample size might limit the generalizability of our findings, and validation in multicenter cohorts is warranted. Additionally, although we integrated bulk, single-cell, and spatial transcriptomic data and confirmed our observations using mIF, multiple technical cross-validation methodologies will further strengthen the conclusions. Although our orthotopic ICC mouse model demonstrated the therapeutic potential of the *SLC2A1* inhibitor BAY-876 by significantly reducing tumor burden and vascular-mediated intrahepatic metastasis, its potential systemic “off-target” effects on tumor cells, immune cells, or endothelial cells remain to be fully elucidated. Consequently, future studies utilizing cell-specific knockout models or pharmacological targeting strategies will be essential to definitively prove this causation. Moreover, while our in vitro and in vivo data provides supportive evidence, the causal link between the hypoxia–tCAFs–VEGF–endothelial cells signaling axis and VI remains an inference at this stage. Furthermore, possible metabolic compensations in CAFs, including increased oxidative phosphorylation following glycolysis inhibition, must be considered when positioning *SLC2A1* as a clinical target. Therefore, in-depth molecular and functional research of the identified candidate targets will contribute to elucidating the precise biological mechanisms of VI formation and progression in ICC.

## Data Availability

The data presented in this study are openly available in [GEO] at [https://www.ncbi.nlm.nih.gov/geo/query/acc.cgi?acc=GSE316921] (accessed on 30 Jan 2026), reference number [GSE316921].

## Supplementary Materials

The following supporting information can be downloaded at: https://www.mdpi.com/article/10.3390/cells15111016/s1, Figure S1. Data quality control and cluster information. (A) Violin plots indicating the detected genes (nFeature RNA), UMIs counted (nCount RNA), the detection rate of ribosomal gene expression (percent rb), and the detection rate of mitochondrial gene expression (percent mt) for each sample. (B) Bar plots showing the distribution of major cell types in each sample. (C) UMAP plots showing the cluster information about Relapse state, differentiation, and tumor size. Figure S2. Characterization of NK/T and myeloid cell subsets in VI+ and VI− ICC. (A) UMAP plot illustrating the NK/T cell subsets. Colors indicate different cell subsets. (B) Dot plot showing the expression of marker genes of each NK/T cell subset. (C) UMAP plot illustrating the myeloid cell subsets. Colors indicate different cell subsets. (D) Dot plot showing the expression of marker genes of each myeloid cell subset. (E,F) Tissue preference of NK/T and myeloid cell subsets in VI+ and VI− ICC tumors, revealed by $R_{o/e}$ analysis. (G) KEGG functional enrichment analysis of TAM_MARCO. Figure S3. Identification and functional analysis of malignant epithelial cells. (A) Heatmap displaying large-scale copy number variations (CNVs) for malignant epithelial cells, with endothelial and NK/T cells included as references. Red represents gains, and blue represents losses. (B) Box plot showing CNV scores of each epithelial cell subset. (C) UMAP plot indicating the distribution of malignant and non-malignant epithelial cell subsets. (D) Tissue preference of malignant epithelial cell subsets in VI+ and VI− ICC tumors, revealed by $R_{o/e}$ analysis. (E) UMAP plot indicating the VI+ and VI− malignant epithelial cells. (F) GSVA analysis of VI+ and VI− malignant epithelial cells. Figure S4. GSVA and survival analysis based on major cell clusters of fibroblast, NK/T, and myeloid cell subsets. (A,B) GSVA heatmap and Kaplan–Meier survival curves on the basis of the FU-iCCA dataset showing the enrichment scores and OS of the 7 major cell clusters. (C,D) GSVA heatmap and Kaplan–Meier survival curves on the basis of the GSE89748 dataset showing the enrichment scores and OS of the 7 major cell clusters. Figure S5. Cell–cell interactions among major fibroblast, NK/T, and myeloid cell subsets. (A) Network plot showing the 7 major cell clusters with differential interaction strength. (B) Comparing the outgoing and incoming interaction strength of 7 major cell clusters in 2D space between VI+ and VI− groups. (C) Chord plot showing changes in the key signaling pathways from tCAF to TAM_MARCO. (D) Bubble plot showing the upregulated ligand–receptor pairs in MIF and FN1 signaling pathways from tCAFs to TAM_MARCO. (E) Chord plot showing changes in the interaction relationships of 7 major cell clusters on the MIF signaling pathway. (F) Expression distribution of the MIF signaling pathway genes in VI+ and VI− groups. Figure S6. Functional analysis of sh*SLC2A1* in ICC-derived CAFs under control and hypoxic conditions. (A) Dot plot showing the expression patterns of candidate genes *PLOD2*, *PTGS2*, and *SLC2A1* across different CAF subtypes. (B,C) Volcano plots showing DEGs between sh*SLC2A1* and control under the same normal or hypoxic conditions. (D,E) GO and KEGG functional enrichment analyses based on the DEGs from the two comparisons. (F) Expression levels of genes related to the TGF-β signaling pathway across the indicated groups. (G) GSEA enrichment analysis of the TGF-β signaling pathway between the sh*SLC2A1* and control groups under hypoxic conditions; Table S1: Clinical information of patients; Table S2: List of signature genes expressed in 6 cell clusters in ICC; Table S3: GO terms of 6 cell clusters; Table S4: Tissue type enrichment of cell clusters revealded by Ro/e; Table S5: GO and KEGG terms of tCAF; Table S6: Proportion of tCAF in immunofluorescence of patient tissue sections; Table S7: GO and KEGG terms of 15 DEGs; Table S8: Summary of primers; Table S9: Expression of genes under hypoxic conditions detected by qPCR; Table S10: Summary of shRNA sequences.

## Abbreviations

ICC, Intrahepatic cholangiocarcinoma; VI, Vascular invasion; TME, Tumor microenvironment; scRNA-seq, Single-cell RNA sequencing; CAF, Cancer-associated fibroblast; GSVA, Gene set variation analysis; KEGG, Kyoto Encyclopedia of Genes and Genomes; GO, Gene Ontology; HUVECs, Human umbilical vein endothelial cells; VEGF, Vascular endothelial growth factor; NC, Negative control; Ace, Acetaminophen; BAY, BAY-876; Min, Minoxidil.
