# Supplementary figures and images for: Single-Cell and Bulk Transcriptomics Uncover the Cellular Ecosystem of Vascular Invasion in Intrahepatic Cholangiocarcinoma
Source: Cells. 2026 May 31;15(11):1016. doi: 10.3390/cells15111016 (PMC13256196; doi:10.3390/cells15111016)

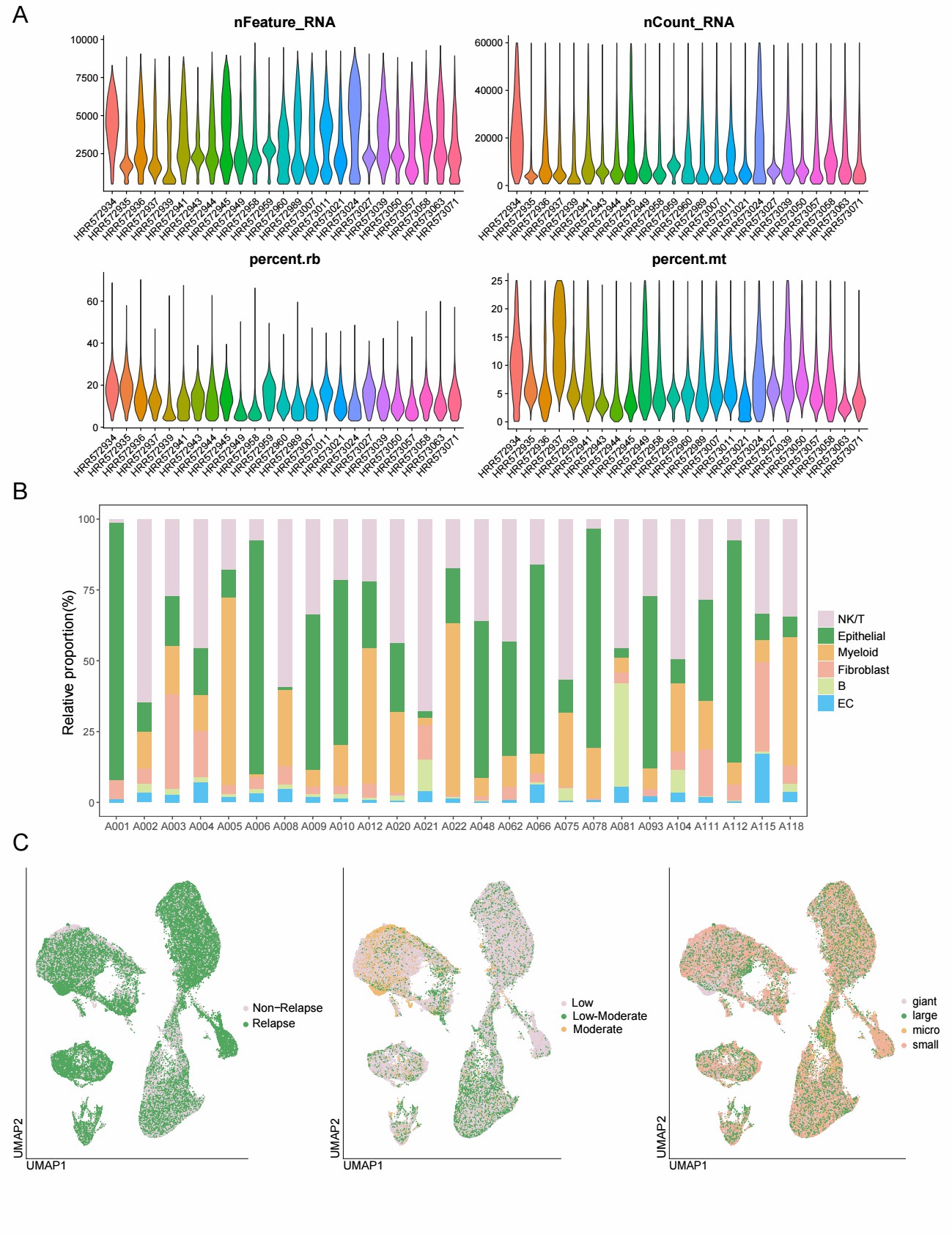

Supplement: Supplementary file 1 [file cells-15-01016-s001.zip › Supplementary Figure S1.jpg]

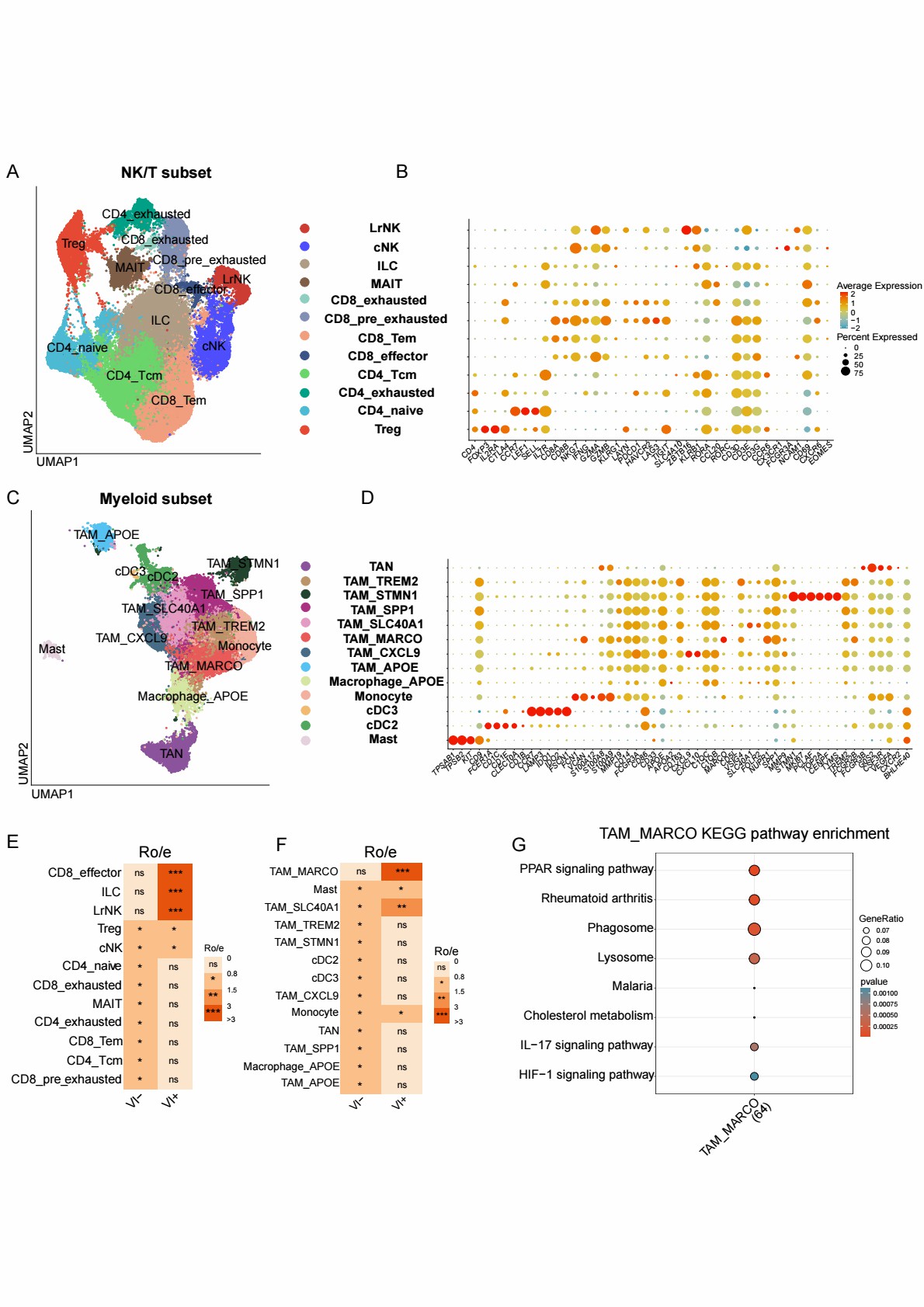

Supplement: Supplementary file 1 [file cells-15-01016-s001.zip › Supplementary Figure S2.jpg]

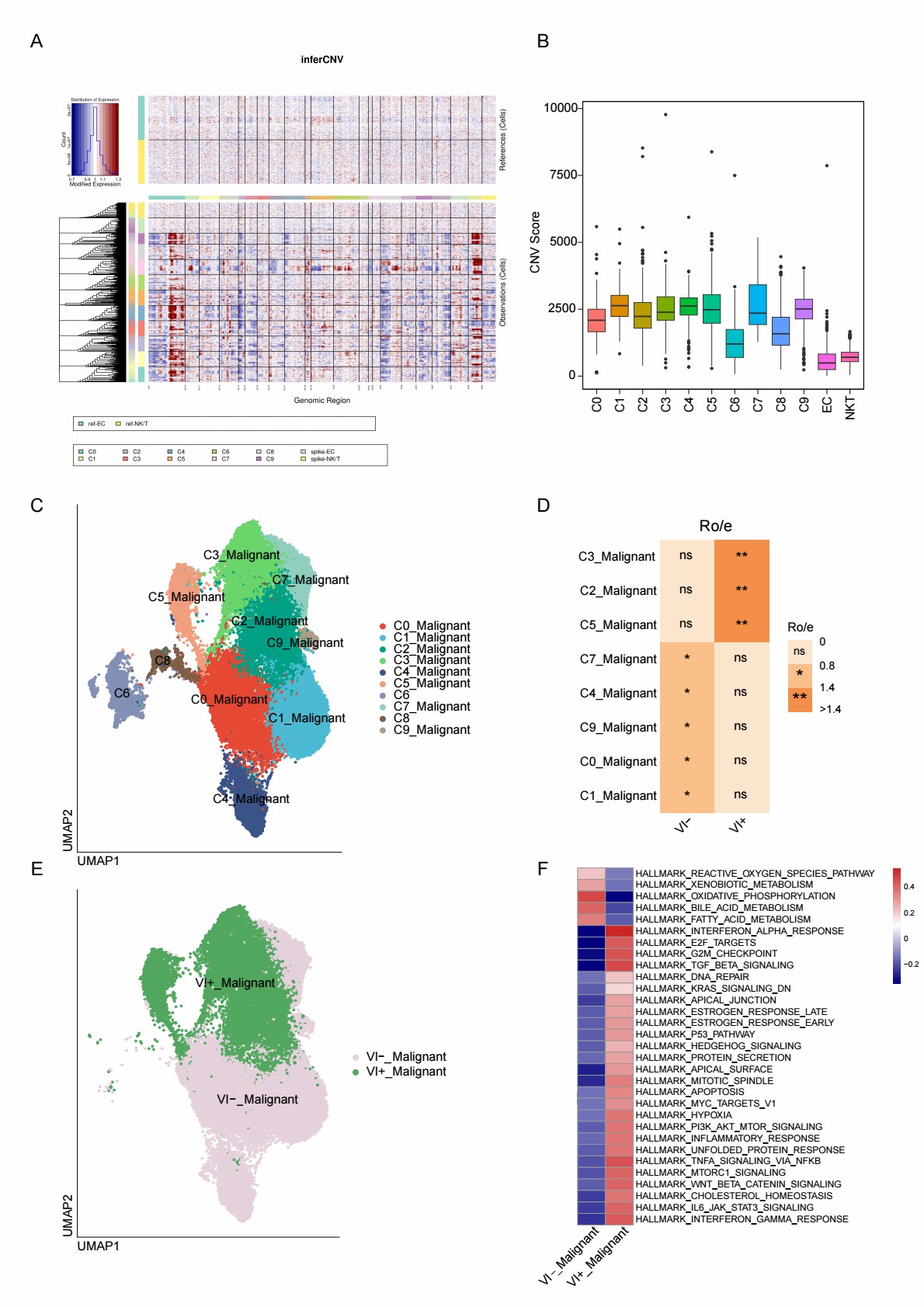

Supplement: Supplementary file 1 [file cells-15-01016-s001.zip › Supplementary Figure S3.jpg]

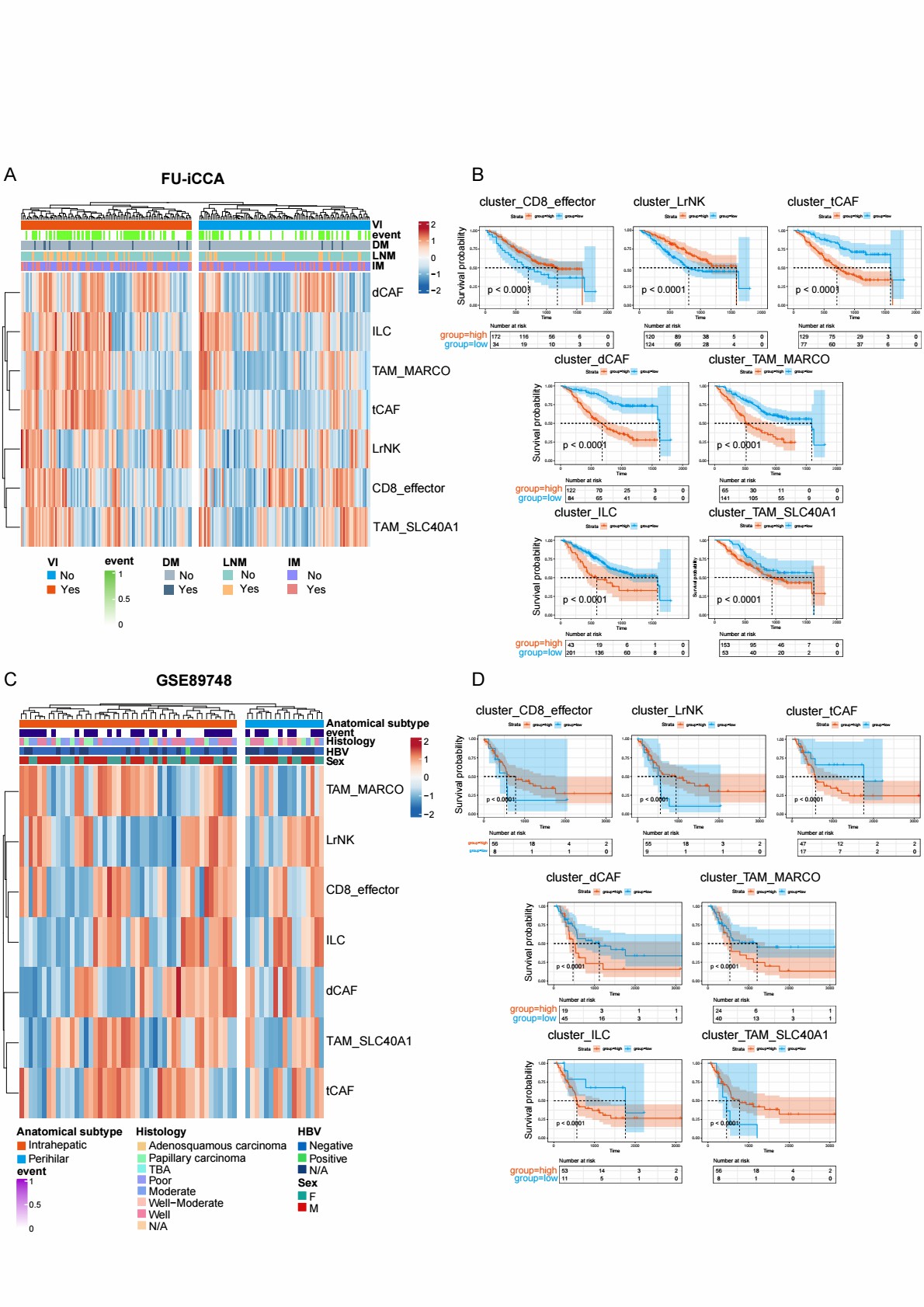

Supplement: Supplementary file 1 [file cells-15-01016-s001.zip › Supplementary Figure S4.jpg]

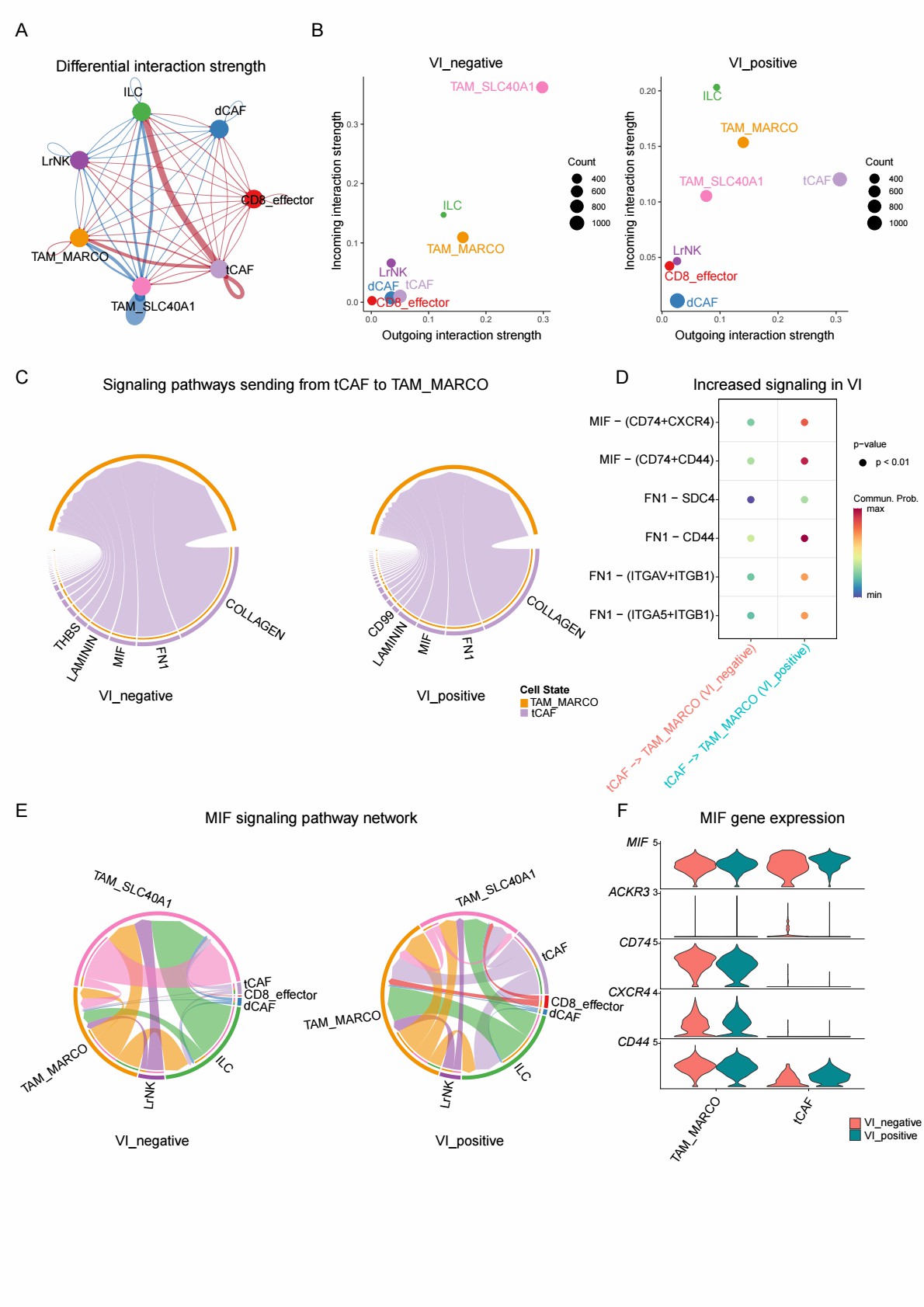

Supplement: Supplementary file 1 [file cells-15-01016-s001.zip › Supplementary Figure S5.jpg]
